# Sources and fate of microplastics in marine and beach sediments of the Southern Baltic Sea—a preliminary study

**DOI:** 10.1007/s11356-017-8419-5

**Published:** 2017-01-25

**Authors:** Bożena Graca, Karolina Szewc, Danuta Zakrzewska, Anna Dołęga, Magdalena Szczerbowska-Boruchowska

**Affiliations:** 1 Department of Marine Chemistry and Environmental Protection, Institute of Oceanography, University of Gdansk, Al. Marszalka Pilsudskiego 46, 81-378 Gdynia, Poland; 2 Department of Inorganic Chemistry, Faculty of Chemistry, Gdansk University of Technology, Gabriela Narutowicza 11/12, 80-233 Gdansk, Poland; 3 Department of Medical Physics and Biophysics, Faculty of Physics and Applied Computer Science, AGH University of Science and Technology, Al. Adama Mickiewicza 30, 30-059 Krakow, Poland

**Keywords:** Microplastics, Pollution, Sediments, Beaches, Southern Baltic, Density separation

## Abstract

Microplastics’ (particles size ≤5 mm) sources and fate in marine bottom and beach sediments of the brackish are strongly polluted Baltic Sea have been investigated. Microplastics were extracted using sodium chloride (1.2 g cm^−3^). Their qualitative identification was conducted using micro-Fourier-transform infrared spectroscopy (μFT-IR). Concentration of microplastics varied from 25 particles kg^−1^ d.w. at the open sea beach to 53 particles kg^−1^ d.w. at beaches of strongly urbanized bay. In bottom sediments, microplastics concentration was visibly lower compared to beach sediments (0–27 particles kg^−1^ d.w.) and decreased from the shore to the open, deep-sea regions. The most frequent microplastics dimensions ranged from 0.1 to 2.0 mm, and transparent fibers were predominant. Polyester, which is a popular fabrics component, was the most common type of microplastic in both marine bottom (50%) and beach sediments (27%). Additionally, poly(vinyl acetate) used in shipbuilding as well as poly(ethylene-propylene) used for packaging were numerous in marine bottom (25% of all polymers) and beach sediments (18% of all polymers). Polymer density seems to be an important factor influencing microplastics circulation. Low density plastic debris probably recirculates between beach sediments and seawater in a greater extent than higher density debris. Therefore, their deposition is potentially limited and physical degradation is favored. Consequently, low density microplastics concentration may be underestimated using current methods due to too small size of the debris. This influences also the findings of qualitative research of microplastics which provide the basis for conclusions about the sources of microplastics in the marine environment.

## Introduction

Since 1950, global plastic production has increased from 1.5 to 311 million tonnes in 2014 (Plastics Europe 2015). Because of increasing production of synthetic polymers and their low biodegradability, plastic pollution has become a serious environmental problem. It has been estimated that every year 4.8–12.7 million tonnes of plastic debris enter the marine environment (Jambeck et al. 2015), and this amount will probably increase by an order of magnitude before 2025 (Jambeck et al. 2015). There are several definitions of microplastics, for example Gregory and Al (2003) defined them as the barely visible particles that pass through a 500 μm sieve but are retained by a 67 μm sieve, while Imhof et al. (2013) classified particles smaller than 1 mm as microplastics. Nowadays, it is widely accepted that plastic items smaller than 5 mm are considered as microplastics (MSFD Technical Subgroup on Marine Litter 2013). Microplastics can enter the marine environment as primary or secondary pollution. Primary microplastics are polymers manufactured in micro-scale, e.g., cosmetics (Zitko and Hanlon 1991) and medicine (Patel et al. 2009) components or raw materials used for plastic production (Turner and Holmes 2011). Secondary microplastics are products of physical (mechanical) and photochemical degradation of bigger plastic fragments (Zbyszewski et al. 2014; Galgani et al. 2015; Koelmans et al. 2015).

It has been proved that microplastics in the marine environment have an impact on organisms of all trophic levels—worms, fishes, sea turtles, birds, and mammals (Wright et al. 2013; Lusher 2015). Many organisms confuse microplastics with food or selectively feed on them in place of food (Moore 2008). Stomach volume occupied by debris may limit optimal food intake induced by a feeling of satiation, and reducing a feeling of hunger (Day et al. 1985) which may reduce the drive to search for food (Hoss and Settle 1990). Subsequently, it may lead to growth rate decrease, decrease of reproductive abilities, and ability to avoid predators (van Franeker 1985; Bjorndal et al. 1994; McCauley and Bjorndal 1999). When the gastrointestinal tract becomes completely blocked or severely damaged with abrasions and ulcers, ingested plastic may lead to rapid death (Kühn et al. 2015; Lusher 2015). Plastic debris in the marine environment contains various hydrophobic pollutants and trace metals at concentrations from ng g^−1^ to μg g^−1^ (Mato et al. 2001; Endo et al. 2005; Rios et al. 2007; Holmes et al. 2014; Rochman et al. 2014; Turner and Holmes 2015). Some of these compounds are added during plastics manufacture, while others are adsorbed from the surrounding seawater. In some marine species organic contaminants can interfere with natural hormone functions, cause mutations and cancer (Neal 1985; Sonnenschein and Soto 1998; Foster 2005). It has been shown that hydrophobic organic contaminants have greater affinity for plastics like polyethylene, polypropylene, and poly(vinyl chloride) than for natural sediments (Teuten et al. 2007, 2009). Reduction of the plastic litter size to the micro-scale presumably enhances their sorption properties and facilitates the transport of the harmful compounds from plastics into the organisms (Staniszewska et al. 2016).

Microplastics are observed globally, not only close to densely populated regions (Browne et al. 2011), but also in remote areas (Barnes 2005; Zarfl and Matthies 2010) and deposition zones (Van Cauwenberghe et al. 2013; Woodall et al. 2014). Sources of microplastics in the marine environment have not been fully examined. Their inputs might be expected from harbors and shipyards, fisheries, wastewater treatment plants, coastal tourism (Stolte et al. 2015), urban runoff (Patters and Bratton 2016), and rivers (Woodall et al. 2014). Their fate in beach and bottom sediments is still not fully understood. It has been known that sediments have the potential to accumulate microplastics (Zalasiewicz et al. 2016), but no clear relationship between microplastics abundance and sediment grain size was noted (Browne et al. 2010; Alomar et al. 2016), like it is observed for organic matter and other contaminants (Zhao et al. 2010; Chakraborty et al. 2015). But aggregation with organic matter might play an important role in microplastics transport (Van Cauwenberghe et al. 2015).

The Baltic Sea is a shallow-water (52 m mean depth), semi-enclosed sea in the northern Europe. The deepest area of the Baltic is 459 m deep (the Landsort Deep), while in the study area the deepest area is the Gdansk Deep (118 m). The Baltic’s catchment is highly urbanized and under constant anthropopressure (human activities, both planned and random, having an impact on the natural environment), particularly from maritime transport and touristic activity. Furthermore, long water exchange (30 years; Franck et al. 1987) favors accumulation of pollutants in the area.

In the present study for the first time, as far as the authors know, morphology, concentration, and fate of microplastics in bottom and beach sediments of the Polish zone of the Southern Baltic Sea were determined. Additionally, main microplastics sources were indicated.

## Materials and methods

### Samples collection

#### Marine bottom sediment samples

**Fig. 1:**
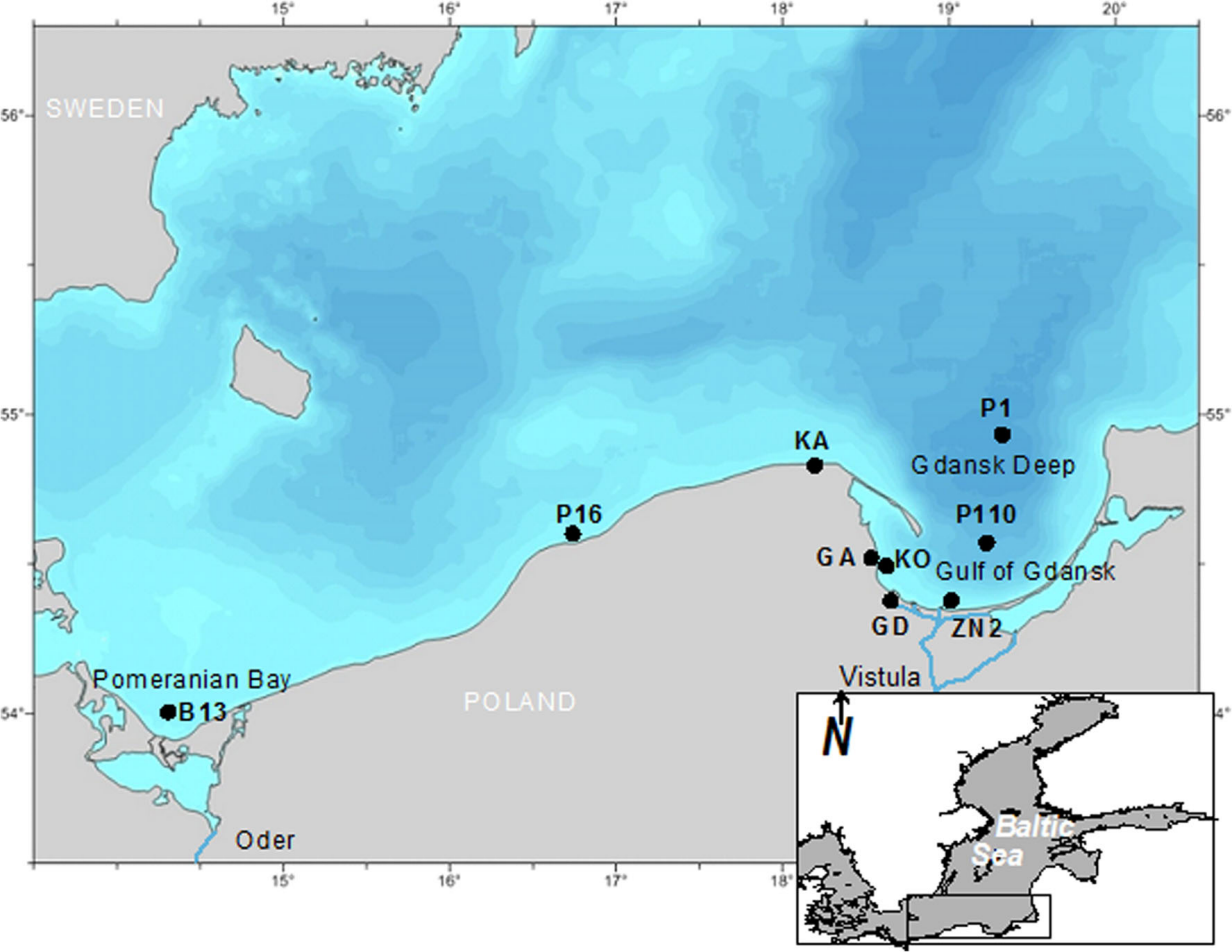
The location of sampling sites in the Southern Baltic Sea

Samples were collected onboard of R/V Baltica at four shallow and two deep-water stations along the Polish coast of the Baltic Sea in April and June 2014 (Fig. 1). Predominant in shallow (11–18 m) regions of the study area (stations: B13, P16, KO, ZN2) sands were collected in two hauls by van Veen grab. Surface layer of the sediment (0–2.5 cm) collected from both hauls was separated using metal ring (10 cm in diameter) and plate, and put into a glass jar. It has been assumed this way of sampling will allow obtaining representative results for sandy sediments of shallow-water coastal zone of the sampling area. In the case of silt/clay deep sea sediments (70–106 m; P1 and P110 stations) van Veen grab has been replaced with Niemistö corer (7 cm diameter) because of visible disturbance of sediments during sampling (using van Veen grab). Five cores were collected at each deep-water station. The ship was hove, so, approximate surface on which samples were collected was about 400 m. Because in studied deep-water area relatively stable conditions occur, it has been assumed that it is a sufficient sampling surface for obtaining representative results for fine grain sediments. Because fine grain sediments in the study area (P1 and P110 stations) in a lesser extent than sands in shallow-water zone (B13, P16, KO, ZN2 stations) undergo re-suspension, bioturbation and irrigation, thinner layer than in the case of sands – 0-1 cm – was analyzed. Linear accumulation rate in the Gdansk Deep and its slope is 1.6 and 1.8 mm y^−1^ , respectively (Graca et al. 2016). Thus, the 0–1 cm surface layer was deposited over the last 5–6 years.

All samples were frozen until analysis.

#### Beach sediment samples

To assess the impact of catchment management on quality and quantity of microplastics on beaches, samples were taken at dune beaches of highly urbanized coast of the Gulf of Gdansk (GD area) and by the open sea, where anthropopressure is weaker (KA area; Fig. 1).

To assess the quality and quantity of microplastics washed ashore, beach sediments samples were taken along a cliff (GA area; Fig. 1) five times at a calm sea state and five times after a storm.

At each studied beach, sediments were collected at 3 km (GD and KA) or 1 km distance (GA) with a subsample every 200 m. Surface (0–2.5 cm) beach sediment was taken using metal ring (10 cm in diameter), plate, and spoon. Then subsamples from the entire distance were integrated in a metal container and stored in a glass jar. Samples were collected from December 2014 to April 2015. This is a season of strong water mixing and limited touristic activity. As a result, collected microplastics were potentially in a greater extent secondary microplastics. The place of microplastics deposition within the beach is probably changeable and strongly depends on waves intensity. To unify samples collection, in the present study samples were always taken from the middle part of the beach. Pilot studies revealed that in the studied period, in this part of the beach accumulates approximately 1.5 times more microplastics than in the part constantly washed by the waves, and 7.5 times more than at the end of the beach.

### Density separation of microplastics

Before selecting a method for microplastics extraction from sediments, a few methods were tested (Thompson et al. 2004; Ng and Obbard 2006; Nuelle et al. 2014) using fine grain sediment samples collected in the field and spiked with a known number of microplastics prepared in a laboratory, and the highest efficiency was obtained using a method described by Thompson et al. (2004) with modifications according to Laglbauer et al. (2014) and some additional changes. Due to smaller grain size in the presented study than in study of Laglbauer et al. (2014), mesh size was reduced (about 5.6 times), and time of shaking and sedimentation were extended (4 times). In effect, 150 g of wet sediment was put into a glass jar. Then 500 ml of concentrated NaCl (about 1.2 g cm^−3^) solution was added. Prepared this way sample was shaken vigorously for 2 min and left for sedimentation. After 2 h, the solution was decanted. The supernatant was filtrated through a 45 μm steel sieve. The material retained on the sieve was washed off into a glass Petri dish using a wash bottle with milli-Q water. For each sample, the procedure was repeated three times.

To avoid contamination by microplastics from the air, the procedure was performed under a clean fume hood. All the glass was covered with aluminum foil.

Blanks consisted of 500 ml of NaCl solution only and were handled the same way as samples. Blanks did not contain microplastics.

### Visual analysis of microplastics and μFT-IR

For visual analysis of microplastics, Nikon SMZ 1000 stereomicroscope with ×10 magnification was used. The microscope was equipped with Schott KL 300 LED polarization light. Microplastics were divided by color and type into fibers, irregular fragments, and plastic films. To distinguish microplastics, a few criteria were considered: no visible cellular structures, clear, homogenous color, equally thick, not taper towards the ends, and three-dimensional bending fibers (Norén 2007). Particles ≤5 mm were considered as microplastics.

Representative microplastic particles were removed from samples using tweezers and stored on microscope slides. Polymer types were determined using μFT-IR reflectance spectroscopy (Thermo Scientific Nicolet Continuμm Infrared Microscope).

### Statistical analysis

Statistical analysis was performed using STATISTICA 12 (StatSoft). The significance of the differences between the obtained results were checked using the Student’s *t* test and Mann-Whitney *U* test (*p* < 0.05).

## Results

### Morphology, concentration, and quality of microplastics in marine bottom sediments

**Fig. 2:**
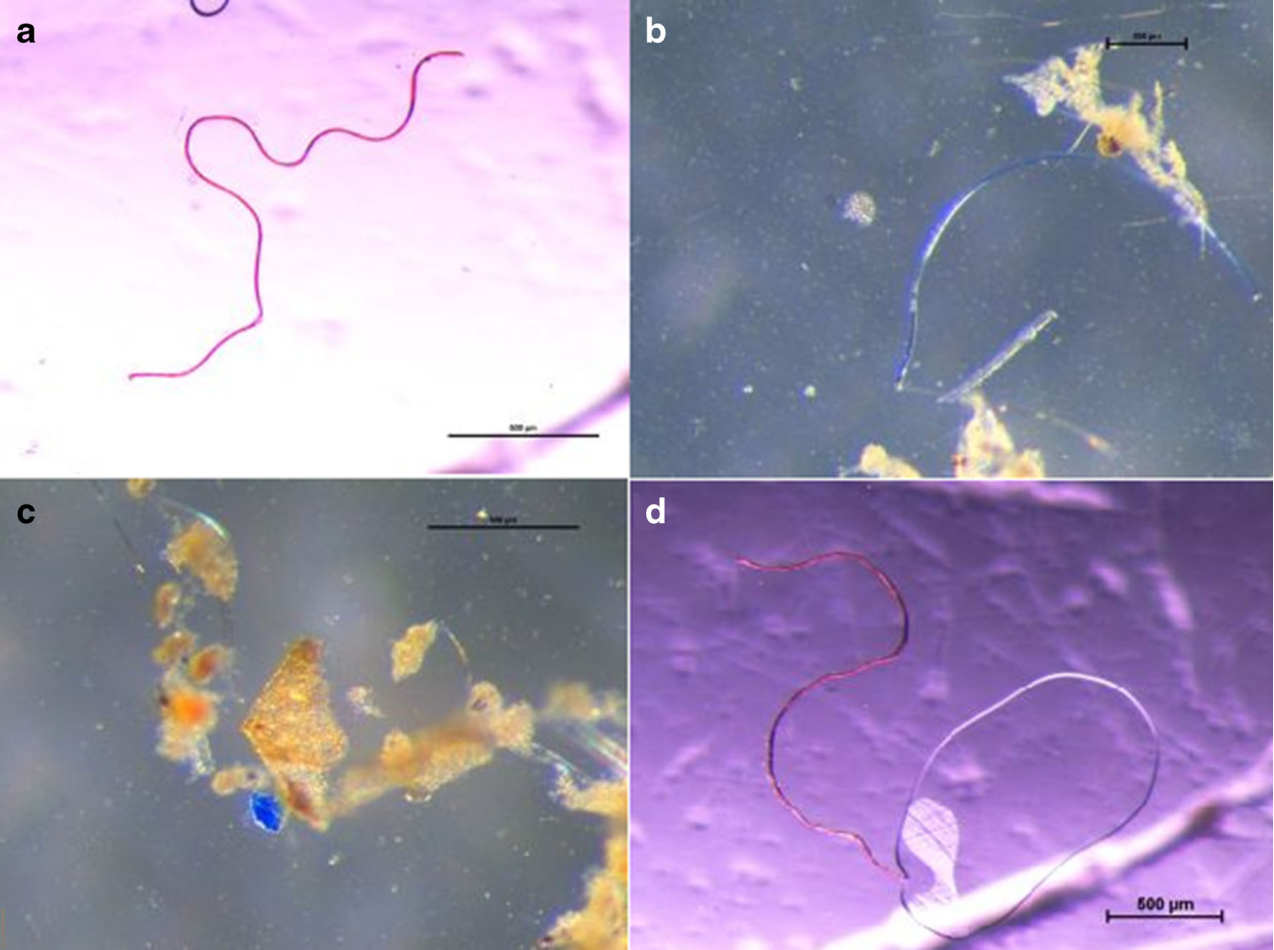
Microplastics extracted from marine bottom sediments and beach sediments of the Southern Baltic Sea. **a** *Red* fiber (GA station at calm sea state). **b** *Blue* fiber (ZN2 station). **c** *Blue* irregular fragment (KO station).**d** *Red* fiber (GA station at calm sea state). *Scale bars* = 500 μm

Fibers were the predominant type of microplastics in marine bottom sediments. Their mean concentration was about 13 times higher compared to concentrations of plastic films or irregular fragments (Table 1). Most of fibers were transparent (73%). Some examples of the different types of microplastics found in the study area has been shown in Fig. 2.

**Table 1:** Concentration of different microplastic types [number of particles kg^−1^ d.w.] in marine bottom sediments and beach sediments of the Southern Baltic Sea

Sediment type	Coast type	Other factors	Station	Fibers	Plastic films	Irregular fragments	Total
marine		Shallow-water coastal zone	ZN2	27	0	0	27
			KO	15	5	5	25
			B13	20	5	5	20
			P16	15	0	0	15
		Deep-water area	P110	3	0	0	3
			P1	0	0	0	0
			Mean ± SD	13 ± 9	1 ± 2	1 ± 2	15 ± 10
			Median	15	0	0	18
beach	dune	Gulf—strong urbanization	GD	43	0	0	43
		Open sea—weak urbanization	KA	25	0	0	25
			Mean ± SD	34 ± 9	0 ± 0	0 ± 0	34 ± 9
			Median	34	0	0	34
	cliff	Calm sea state	GA	53	0	0	53
				46	0	0	46
				39	0	0	39
				53	0	0	53
				46	0	7	53
			Mean ± SD	47 ± 5	0 ± 0	1 ± 3	49 ± 6
			Median	46	0	0	53
		After a storm	GA	25	6	0	31
				25	0	0	25
				26	0	6	32
				25	6	6	37
				32	0	0	32
			Mean ± SD	23 ± 3	2 ± 3	2 ± 3	31 ± 4
			Median	25	0	0	32

**Fig. 3:**
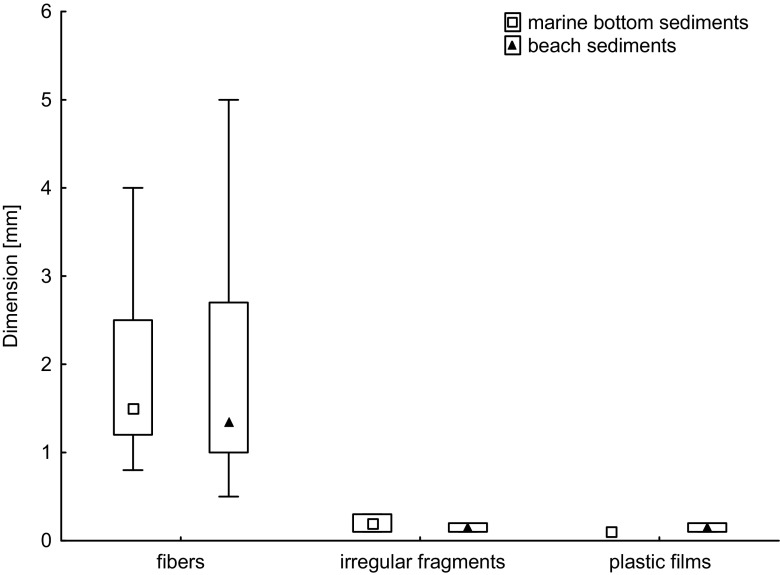
Dimensions (mm) of different microplastic types extracted from marine bottom sediments and beach sediments of the Southern Baltic Sea

Dimensions of separated microplastics ranged from 0.1 to 4.0 mm (the length of fibers or in a case of plastic films and irregular fragments—the longest edge—were considered), and relatively small (0.1–2.0 mm) microplastics were predominant (64%; Fig. 3).

Microplastics concentration ranged from 0 to 27 particles kg^−1^ d.w. and decreased with increasing distance from the shore (Table 1, Fig. 1). The most abundant in microplastics were sandy sediments collected close to the Vistula river outlet (ZN2 station, 27 microplastics kg^−1^ d.w.). On the slope of the Gdansk Deep (P110 station), microplastics concentration was nine times lower, and in sediment from the deepest area, the Gdansk Deep (P1 station) none microplastics were observed. The westernmost Pomeranian Bay (B13 station) contained 20 microplastics kg^−1^ d.w., whereas midmost shallow-water part of the Southern Baltic (P16 station) contained 15 microplastics kg^−1^ d.w.

**Fig. 4:**
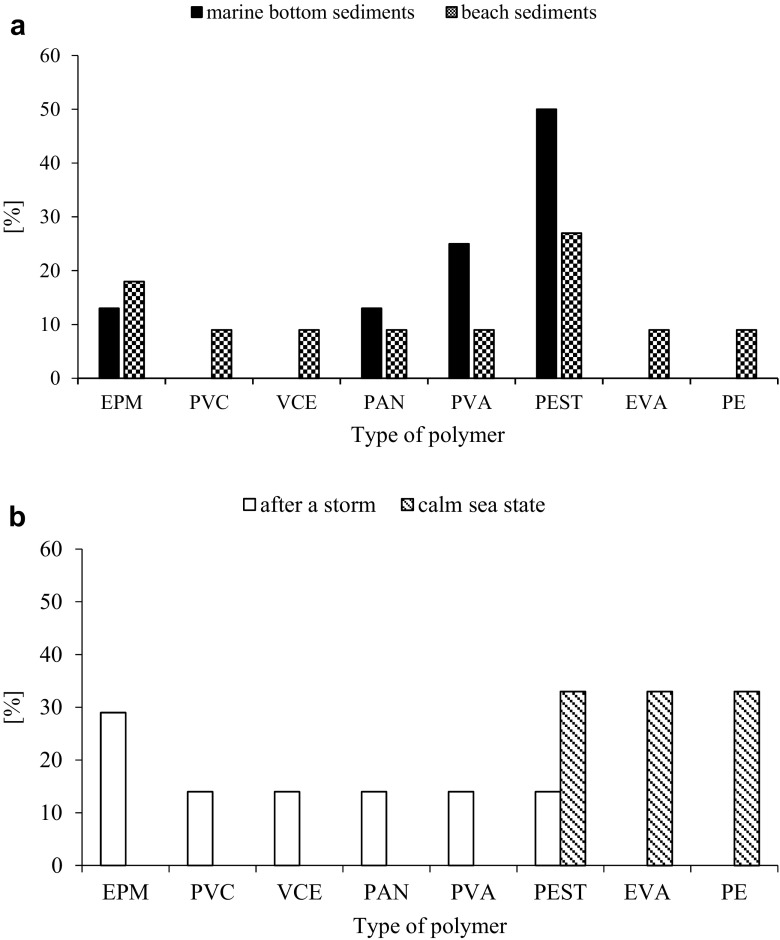
Percentage of different polymer types **a** in marine bottom sediments and beach sediments of the Southern Baltic Sea, and **b** at the cliff coast of the Southern Baltic Sea depending on the seawater dynamical conditions

Four types of polymers were identified (Fig. 4a). The most numerous polymers in marine bottom sediments were PEST (50%) and PVA (25%; Fig. 4a).

### Morphology, concentration, and quality of microplastics in beach sediments

Similarly as in the case of bottom sediments, in beach sediments fibers were the predominant type of microplastics. Only a few or none plastic films (pink and transparent) or irregular fragments (transparent, blue, red) were observed (Table 1). Transparent, red, blue, and green fibers accounted for 76, 6, 17, and 1% of all fibers respectively.

Dimensions of separated microplastics ranged from 0.1 to 5.0 mm (the length of fibers or in the case of plastic films and irregular fragments—the longest edge—were considered), and relatively small (0.1–2.0 mm) microplastics were predominant (82%; Fig. 3).

Microplastics concentration in beach sediments ranged from 25 to 53 kg^−1^ d.w. (median value 38, mean value 39 ± 10 kg^−1^ d.w.; Table 1) and was statistically significantly higher (Mann-Whitney *U* test, *p* = 0.03) than microplastics concentration in marine bottom sediments (median value 18; mean value 15 ± 10 kg^−1^ d.w., Table 1).

Sediment sampled from the dune beaches along the gulf (GD area) at a calm sea state contained 43 microplastics kg^−1^ d.w. In the same conditions, sediment from the beach by the open sea where the degree of urbanization is weaker (KA area) contained 25 microplastics kg^−1^ d.w.

In the cliff area (GA station), the mean value of microplastics concentration during a calm sea state was 49 ± 9 particles kg^−1^ d.w., whereas after a storm, it was 31 ± 4 kg^−1^d.w (Table 1). The differences were statistically significant (Student’s *t* test, *p* = 0.00).

Within beach sediments, eight types of polymers were identified (Fig. 4a). The most common polymers were PEST (27%) and EPM (18%). In the cliff area, qualitative composition of microplastics was changing depending on the seawater dynamics (Fig. 4b). At the times of a calm sea state, three types of polymers were identified: EVA, PE, and PEST. After a storm, six polymer types were distinguished: EPM, PEST, PVC, VCE, PAN, and PVA.

## Discussion

### Factors determining microplastics concentration in marine bottom sediments and beach sediments

Microplastics concentration in marine bottom and beach sediments in different regions varies significantly (Table 2). In huge variety of coast (Ng and Obbard 2006; Claessens et al. 2011; Lee et al. 2013; Laglbauer et al. 2014; Stolte et al. 2015) and marine bottom types (Norén 2007; Claessens et al. 2013; Vianello et al. 2013), different factors can be crucial for abundance of plastic pollution. Marine bottom sediments were sampled mostly in shallow waters, harbors, or offshore (Norén 2007; Claessens et al. 2013; Vianello et al. 2013). In the case of beach sediments, samples were collected between the high and low tide mark (Laglbauer et al. 2014), on the high watermark, in intertidal, subtidal (Claessens et al. 2011), on the strandline (Lee et al. 2013), on the drift line above seawater level (Stolte et al. 2015), or 0.5 m from the tideline (Ng and Obbard 2006). Obtained in this study results are lower than in the other regions of Europe (Table 2). This difference could be affected by the shoreline cover. Almost 2/3 of Polish coast is a wide dune coast covered with pine forest (Zawadzka-Kahlau 1999). This is probably a natural obstacle for debris in the way from the land to the sea. Rocky coast, skerries, fiords or anthropogenically altered coasts, e.g., harbors and lagoons, potentially favor transport of debris to the marine environment. On the other hand, comparison of observed regional differences in microplastics pollution is difficult due to the lack of unitary microplastics size definition, different sampling methods, filter/mesh size used in the analysis, or data estimation on various mass/volume/area units. For microplastics recovery, filter pores/mesh ranging from 0.7 to 1000 μm were used (Table 2). Microplastics abundance is given for example per 1 kg d.w. or 100 ml of sample (Table 2).

**Table 2:** Comparison of microplastics (MPs) concentration with different sampling methods and analytical approach between the Southern Baltic Sea and other regions of the world. Sample mass means dry weight mass

Area	Part	Location	Extracting agent (g cm^−3^)	Filter/mesh size(μm)	Sample mass/volume/area	Sampling site	MPs	References
Baltic Sea	S	Marine	NaCl 1.2	45	1 kg	Gulfs and offshore	0–27	present study
		Beach	NaCl 1.2	45	1 kg	Middle	25–53	
	W	Beach	CaCl_2_ 1.30–1.35	55	1 kg	Drift line above seawater level	0–14	Stolte et al. 2015
North Sea	N	Marine	NaCl 1.2	2	100 ml	harbors and offshore	2–332	Norén 2007
	S	Marine	NaCl 1.2	38	1 kg	Harbors and offshore	72–116	Claessens et al. 2011
		Beach	NaCl 1.2	38	1 kg	High watermark, intertidal, subtidal	49–156	
Adriatic Sea	N	Marine	NaCl 1.2	0.7	1 kg	Shallow waters; freshwater, agriculture, urban, industrial inputs	672–2175	Vianello et al. 2013
	E	Beach	NaCl 1.2	250	1 kg	Between high and low tide mark, infralittoral	133–444	Laglbauer et al. 2014
Japan Sea	S	Beach	-	1000	m^2^	Along strandline	2–92	Lee et al. 2013
Pacific Ocean	SE	Beach	NaCl 1.2	1.6	1 kg	0.5 m from tideline	0–8	Ng and Obbard 2006

The factors which probably contribute to different microplastics concentration at beach stations located by the open sea (KA area) and by the gulf (GD, GA area) are tourism and urbanization. Beaches along the gulf are more frequently visited by tourists than those by the open sea. What is more, the beaches located by the Gulf of Gdansk are in the immediate vicinity of two big cities—Gdansk and Gdynia. Similar conclusions were reached by Stolte et al. (2015), who observed higher microplastics concentration in sediments from touristic Rostock beach. In our study, conducted in the Southern Baltic, beach sediment samples were taken in winter; however, if they were taken in summer during the touristic season, the discrepancy in microplastics concentration between the open sea area and the gulf probably would be greater.

Plastic litter from the land can be transported to the open sea. Deep sea sediments are a place of potential microplastics accumulation (Woodall et al. 2014). This process depends on many complex factors, such as water depth, sea bottom topography, organisms present in the area, and hydrodynamical conditions (Woodall et al. 2014). In the present study, in the deepest examined area (the Gdansk Deep, P1 station) microplastics were not observed. The Gdansk Deep reaches a maximum depth of 118 m. At 70 m, a permanent halocline is present. The halocline could probably limit accumulation of microplastics in sediment due to large increase in water density. At the Gdansk Deep slope (P110 station), where sediment contained microplastics, conditions are more dynamic, and the halocline is unstable. Still, this hypothesis requires confirmation in research. Furthermore, lack of microplastics in sediment samples from a place of their potential accumulation could be a consequence of the method used in the study. Fine grain size and organic matter in silt/clay covering this region impede the density separation and visual analysis. During density separation, fine sediment grains are supported by surface tension, therefore, unambiguous separation of microplastics and sediment particles is impeded (Stolte et al. 2015). It is necessary to use more sophisticated techniques for microplastics extraction from fine sediments.

Freshwater inflow is considered as a source of microplastics (Browne et al. 2011). Into the Gulf of Gdansk and the Pomeranian Bay, large quantities of freshwater are discharged by the Vistula and the Oder rivers. The Vistula is the second biggest river in the Baltic catchment with the average flow rate of 1081 m^3^ s^−1^. The Oder has almost two times lower flow rate than the Vistula (572 m^3^ s^−1^) (HELCOM 2004). However, a factor reducing microplastics concentration in the Gulf of Gdansk and the Pomeranian Bay might be their open character. As a result, total water exchange in the Gulf of Gdansk takes about 2 weeks (Witek et al. 2003) and in the Pomeranian Bay about 3 weeks (Jost and Pollehne 1998). Despite “this cleansing” activity, proximity of urbanized areas, riverine inflow, and intensive exploitation (fisheries, tourism, etc.) probably contributed to increased concentration of microplastics in marine bottom sediments of examined bays (the Pomeranian Bay—20 microplastics kg^−1^ d.w., the Gulf of Gdansk—25 microplastics kg^−1^ d.w.) relative to sediments from the open sea (the Gdansk Deep and its slope—0 and three microplastics kg^−1^ d.w.; Table 1).

Due to potential large spatial variability of microplastics concentration in the environment, sample representativeness is very important (Gregory and Al 2003). In the present study, marine bottom sediment samples were taken on relatively small area, and their representativeness for a bigger area should be treated with caution. However, the fact that microplastics were observed in sediments collected from small areas suggests that the marine sediment in the study area might be highly polluted with this type of debris.

In the studies conducted on beach sediments using similar to presented research filtration system, domination of relatively big, 1–5 mm in diameter, microplastics were observed (Martins and Sobral 2011; Laglbauer et al. 2014). In the present study, predominant were particles of dimensions 0.1–2.0 mm. Average dimensions of fragments and films were smaller than fibers (0.18 and 0.13 mm, respectively). The length of fibers varied from relatively short (0.5 mm) to relatively long (5.0 mm); Fig. 3), and long fibers (>3 mm) were rare (about 1 fiber in 10 was >3 mm long). This indicates fragmentation of microplatics in the marine environment. Small size is a key factor determining microplastics bioavailability to small organisms of lower trophic levels and is a great threat especially for filter feeders (Wright et al. 2013). Due to increasing distance from a potential source of plastic pollution and differences in rate of their degradation in the water and on the land, a significant difference in microplastics size extracted from marine bottom sediments and from beach sands was expected. But the numbers of fragments and plastic films found in the study area were too small for statistical analysis, and in the case of fibers, the size did not differ statistically (Student’s *t* test *p* = 0.26; Fig. 3). It could be affected by the analytical method. Potentially smaller microplastics could not be retained on a sieve with 0.45 μm mesh size, and those that were managed to separate could be covered with organic matter and sediment grains, thus it was difficult to separate them manually with tweezers.

### Sources and fate of microplastics in the marine environment

An important role in microplastics delivery to the marine environment may be played by wastewater treatment effluents. In this case PEST, which contributes in 78% to the world’s synthetic fabrics production, is a predominant type of microplastics pollution (Browne et al. 2011; Browne 2015). Likewise in the presented study, Woodall et al. (2014) in the North Sea observed predominance of PEST fibers in marine bottom sediments (53.4%). On the other hand, a recent study revealed that the majority of microplastic fragments and fibers are removed during the early skimming and settling stages of primary treatment, therefore discharges from wastewater treatment facilities may be contributing only minimally to microplastics load to environment (Carr et al. 2016). Instead of wastewater effluents, a greater role in microplastics delivery to the marine environment may be played by illegal trash disposal, urban runoff, and aerial transport (Patters and Bratton 2016). For example, Plastics Team (2016) noticed that the mean number of ingested by sunfish microplastics positively correlated with the area of major roads located within 40 km distance from the coast. Other polymers observed in relatively high number in the study area are used in shipbuilding (PVA) and packaging (EPM). Therefore, qualitative research of microplastics in the study area indicate that besides wastewater effluents, maritime transport and tourism are potentially also important sources of microplastics pollution to the southern part of the Baltic Sea.

Greater qualitative differentiation of microplastics in beach than marine bottom sediments of the study area may indicate that plastic debris in a greater extent originates from the land than e.g., maritime transport or fisheries. Similar situation is observed also in other regions of the world, e.g., Australia, United Arab Emirates, Chile, Portugal, Philippines, USA, Mozambique, and UK (Browne et al. 2011). On the other hand, it can be assumed that differences in polymer densities are also responsible for it. More debris of density higher than seawater (1.02 g cm^−3^) can settle on the seabed. Low density plastics float on seawater. Residence time (floating) of litter in water column depends on the type of plastic and physicochemical conditions of water (Muthukumar et al. 2011). In the present study, most of plastics separated from marine bottom sediments have greater density than seawater (Table 3). It means they can sink and reach sediments. Because many organisms ingest microplastics, their removal from water column can occur with fecal pellets (Wright et al. 2013) regardless of polymer density. The presence of less dense plastics in marine bottom sediments can be also an evidence of biofouling. After short exposition time (about 1 week), biofilm develops on plastic litter; density of litter increases, and before 3 weeks, it sinks (Lobelle and Cunlife 2011). Besides biofouling, aggregation with organic matter, and subsequent sedimentation could be an important factor affecting the presence of low density microplastics in marine bottom sediments. As a result, also polymers of density lower than 1.02 g cm^−3^, such as PE, EPM, and EVA, have been identified in bottom sediments of the study area.

**Table 3:** Density (g cm^−3^) of polymers observed in marine bottom sediments and beach sediments of the Southern Baltic Sea (Plastics Team, 2016)

Polymer	Density (g cm^−3^)
PE	0.89–0.98
EPM	0.92–1.0
EVA	0.93–0.94
PAN	1.14–1.17
PVA	1.17–1.20
PVC	1.19–1.35
PEST	1.39–1.44

Cliff coast is a potentially good study area of secondary microplastics pollution due to the protection that it provides from direct pollution from the land. Polymers present at the cliff coast after a storm have density lower than those observed in beach sediments at a calm sea state (Fig. 4b; Table 3). Furthermore, in marine bottom sediments, polymers of density > 1 g cm^−3^ were predominant, while in beach sediments, predominant were polymers of density < 1 g cm^−3^. This indicates that in the coastal zone plastic debris, especially those of low density, are constantly washed ashore and offshore which potentially favor their fragmentation. Due to insufficient quantities of the material, it was impossible to check with statistical test if the size of microplastics was related with their density, but the majority of analyzed bigger size microplastics were made of higher density polymers. In effect, many of low density polymers probably could not be detected using Thompson’s method or other methods applying density separation because the particles were too small. Therefore, it can be concluded that current research methods of microplastics concentration act selectively. They allow extraction of bigger size microplastics which mostly include relatively high density debris because their fragmentation in the marine environment is slower than low density plastic debris. It is essential when we conclude about the sources of microplastics pollution to the marine beach and bottom sediments on the basis of qualitative research of microplastics extracted using density separation methods. Such selectivity may lead to incorrect conclusions. Additionally, it should be noted that according to Hildago-Ruz et al. (2012), less dense plastics float in concentrated saline NaCl solution (1.2 g cm^−3^), while more dense ones float only in sodium polytungstate solution (1.4 g cm^−3^). But in the presented study, microplastic fibers made of high density polymers such as PEST (1.4 g cm^−3^) has been identified. This indicates that density separation with NaCl solution probably in a greater extent allows separation of plastic fibers than plastics of other shapes regardless of their density. Therefore, it can be suspected that the pollution of the study area by higher density microplastics other than fibers (e.g., films and irregular fragments) may be underestimated.

## Conclusions

Obtained results indicate that important factor affecting microplastics (≤5 mm) concentration in beach sediments of the Southern Baltic Sea might be urbanization degree of the closest areas of the catchment. High concentrations (up to 43 particles kg^−1^ d.w.) were typical for dune beaches of strongly urbanized bay. Visibly lower concentrations (25 particles kg^−1^ d.w) were observed at the open sea beaches where urbanization is weaker.

In bottom sediments, microplastics concentration (0–27 particles kg^−1^ d.w.) was visibly lower compared to beach sediments (up to 53 particles kg^−1^ d.w.) and decreased from the shore to the open deep sea regions. Furthermore, obtained results indicate that strong influence on microplastics concentration in marine bottom sediments has probably water exchange rate and water column density stratification connected to halocline occurrence.

In comparison with other coastal areas, concentration of microplastics in beach and bottom sediments of the study area are relatively low. Although, such comparison should be treated with caution due to different research methods of microplastics concentration.

The lower size limit of microplastics found in marine bottom and beach sediments was 0.1 mm. The most frequent microplastics dimensions ranged between 0.1 and 2.0 mm, and transparent fibers were predominant in the study area. However, there is a possibility that density separation with NaCl solution favors separation of fibers compared to plastics of other shapes, but this hypothesis requires confirmation in research.

Qualitative research of microplastics revealed presence of eight types of synthetic polymers. PESTs which are popular fabrics component were the most common type of microplastics in both marine bottom (50%) and beach sediments (27%). Additionally, PVA used in shipbuilding as well as EPM used for packaging were numerous in marine bottom (25% of all polymers) and beach sediment (18% of all polymers), respectively. Taking into account the application of polymers, which the most common microplastics were made of, it can be concluded that their important source in beach and marine bottom sediments in the study area could be wastewater treatment plants effluents, maritime transport, and tourism.

Polymer density seems to be an important factor influencing microplastics circulation between the beach and the sea. The research conducted in the cliff area at a calm sea state and after storms indicate that low density plastic debris probably recirculate between beach sediments and seawater in a greater extent than higher density debris. Therefore, their deposition is potentially limited and physical degradation is favored. Consequently, low density microplastics concentration in beach and bottom sediments may be underestimated using current methods of microplastics separation due to their potentially very small size. This influence also findings of qualitative research of microplastics which provide the basis for conclusions about their sources in beach and bottom sediments.

## Abbreviations

EPM: Poly(ethylene-propylene)
EVA: Poly(ethylene-vinyl acetate)
PAN: Polyacrylonitrile
PE: Polyethylene
PEST: Polyester
PP: Polypropylene
PVA: Poly(vinyl acetate)
PVC: Poly(vinyl chloride)
VCE: Poly(vinyl chloride-ethylene)
